# Evaluation of the Revised Criteria for Biological and Clinical Staging of Alzheimer Disease

**DOI:** 10.1001/jamaneurol.2025.1100

**Published:** 2025-05-19

**Authors:** Alexa Pichet Binette, Ruben Smith, Gemma Salvadó, Pontus Tideman, Isabelle Glans, Danielle van Westen, Colin Groot, Rik Ossenkoppele, Erik Stomrud, Piero Parchi, Henrik Zetterberg, Kaj Blennow, Niklas Mattsson-Carlgren, Shorena Janelidze, Sebastian Palmqvist, Oskar Hansson

**Affiliations:** 1 Clinical Memory Research Unit, Department of Clinical Sciences Malmö, Faculty of Medicine, Lund University, Lund, Sweden; 2 Department of Physiology and Pharmacology, Université de Montréal, Montréal, Quebec, Canada; 3 Centre de Recherche de l’Institut Universitaire de Gériatrie de Montréal, Montréal, Quebec, Canada; 4 Memory Clinic, Skåne University Hospital, Malmö, Sweden; 5 Diagnostic Radiology, Institute for Clinical Sciences Lund, Lund University, Sweden; 6 Image and Function, Skåne University Hospital, Lund, Sweden; 7 Alzheimer Center Amsterdam, Neurology, Vrije Universiteit Amsterdam, Amsterdam University Medical Center, Amsterdam, the Netherlands; 8 IRCCS, Istituto delle Scienze Neurologiche di Bologna (ISNB), Bologna, Italy; 9 Department of Biomedical and Neuromotor Sciences, University of Bologna, Bologna, Italy; 10 Department of Psychiatry and Neurochemistry, Institute of Neuroscience and Physiology, The Sahlgrenska Academy, University of Gothenburg, Mölndal, Sweden; 11 Clinical Neurochemistry Laboratory, Sahlgrenska University Hospital, Mölndal, Sweden; 12 UK Dementia Research Institute at UCL, London, United Kingdom; 13 Department of Neurodegenerative Disease, UCL Institute of Neurology, London, United Kingdom; 14 Hong Kong Center for Neurodegenerative Diseases, Hong Kong, China; 15 Wisconsin Alzheimer’s Disease Research Center, University of Wisconsin School of Medicine and Public Health, University of Wisconsin-Madison, Madison, Wisconsin; 16 Paris Brain Institute, ICM, Pitié-Salpêtrière Hospital, Sorbonne University, Paris, France; 17 Neurodegenerative Disorder Research Center, Division of Life Sciences and Medicine, and Department of Neurology, Institute on Aging and Brain Disorders, University of Science and Technology of China and First Affiliated Hospital of USTC, Hefei, PR China; 18 Department of Neurology, Skåne University Hospital, Lund University, Lund, Sweden; 19 Wallenberg Center for Molecular Medicine, Lund University, Lund, Sweden

## Abstract

**Importance:**

While clinical disease stages remained largely unchanged in the 2024 update of the Alzheimer disease (AD) criteria, tau–positron emission tomography (PET) was introduced as a core biomarker and its spatial extent was incorporated into the revised biological stages of the disease. It is important to consider both the clinical and the biological stages and understand their discrepancies.

**Objective:**

To compare individuals who have discrepant biological and clinical stages with those who have congruent stages in terms of copathologies, comorbidities, and demographics.

**Design, Setting, and Participants:**

Participants were from the Swedish BioFINDER-2 (inclusion from 2017 through 2023) and the Alzheimer’s Disease Neuroimaging Initiative (ADNI) (inclusion from 2015 through 2024). BioFINDER-2 included a prospective population-based (cognitively normal [CN] older adults) and memory clinic–based cohort (participants with subjective cognitive impairment [SCD], mild cognitive impairment [MCI], and dementia). ADNI included a volunteer-based sample. All participants who were amyloid-β positive and had undergone tau-PET were included. In BioFINDER-2, 838 participants of a total of 1979 were included, and of 927 with tau-PET in ADNI, 380 were included.

**Exposures:**

The clinical (CN to dementia) and biological (based on PET; initial [amyloid-β-positive only] to advanced [amyloid-β-positive, elevated, and widespread tau]) stages from the revised AD criteria.

**Main Outcomes and Measures:**

Cross-sectional measures of neurodegeneration (cortical thickness, TAR DNA-binding protein 43 [TDP-43] imaging signature, neurofilament light [NfL]), α-synuclein cerebrospinal fluid status, plasma glial fibrillary acidic protein, white matter lesions, infarcts, microbleeds, comorbidities, and demographics.

**Results:**

There were 838 BioFINDER-2 participants (mean age, 73.9 [SD, 7.3] years; 431 women [51%]; 407 men [49%]) and 380 ADNI participants (average age, 72.9 [SD, 7.0] years; 194 women [51%]; 186 mean [49%]) included. In BioFINDER-2, 37.7% of the sample had congruent biological and clinical stages (reference group), 51.3% had more advanced clinical impairment compared with their clinical stage (clinical > biological) and 11.0% had the opposite (biological > clinical). The main differences were between the reference group and the clinical > biological group: the latter participants were more often positive for α-synuclein pathology, had higher NfL levels, greater TDP-43–like atrophy, and higher burden of cerebral small vessel disease lesions (all false discovery rate *P* < .05). The only difference between the biological > clinical and the reference group was that the former had less neurodegeneration (thicker cortex; all false discovery rate *P *< .001). The main results were replicated in the independent ADNI cohort, where congruent 56.1% of participants had biological and clinical stages; 36.1% were in the category clinical > biological, and 7.9% in biological > clinical.

**Conclusions and Relevance:**

Copathologies play an important role in symptom severity in individuals who harbor less tau-tangle pathology than expected for their clinical impairment. These results highlight the importance of measuring non–AD biomarkers in patients with AD with worse cognitive impairment than expected based on their biological stage, which could impact the clinical diagnosis and prognosis.

## Key Points

**Question:**

What are the main differences between individuals with concordant vs discordant clinical and biological stages of Alzheimer disease (AD), based on the revised criteria for diagnosis and staging of AD?

**Findings:**

In the Swedish BioFINDER-2 and the Alzheimer Disease Neuroimaging Initiative cohorts, participants who had more advanced clinical impairment compared with their underlying biological AD stage more often presented pathologies related to α-synuclein pathology, cerebral small vessel disease, and more advanced neurodegeneration compared with those who had matching clinical and biological stages.

**Meaning:**

A worse clinical than biological stage of AD may indicate that other pathologies or processes are contributing to some or most of the displayed symptoms, which could impact the clinical diagnosis and prognosis.

## Teaser

This study compares individuals who have discrepant biological and clinical stages with those who have congruent stages in terms of copathologies, comorbidities, and demographics.

## Introduction

Alzheimer disease (AD) begins with the accumulation of amyloid-β (Aβ) plaques in the brain, which is followed years later by the deposition of tau tangles in the medial temporal lobe and neocortex. This pathophysiological cascade typically starts while individuals are still asymptomatic.^1,2^ In 2018, the National Institute on Aging and the Alzheimer Association introduced a research framework that defined AD based on its underlying pathology.^3^ Recently, revised criteria for the diagnosis and staging of AD proposed by the Alzheimer Association have been published, incorporating the latest advances in biomarkers.^4^ In this latest edition, AD biomarkers are categorized into core 1 and core 2 biomarkers. The former include biomarkers that become abnormal in the early stages of the disease and reflect Aβ pathology or soluble phosphorylated tau levels, like Aβ–positron emission tomography (PET), cerebrospinal fluid (CSF) Aβ42/40, or plasma p-tau217. The latter include those that become abnormal in later stages of the disease and reflect insoluble aggregates of tau tangles. Tau-PET imaging has been validated as the most reliable core 2 biomarker and is the only biomarker that can currently be used to discriminate between biological stages, which is the focus of the current project. The biological staging requires Aβ-positivity and is composed of 4 stages with progressively greater spatial extent of tau-PET with each stage. The biological staging is distinct from the clinical staging, which is operationalized by a 1 to 6 clinical severity scheme. The clinical staging represents a continuum in which early stages represent no cognitive impairment, intermediate stages represent mild impairment, and severe impairment is present in late stages. Given that cognitive decline can result from various conditions, it is essential to consider both the AD biological and clinical staging along the 2 continuums. If the clinical stage is more advanced than the biomarker stage, it is possible that other brain pathologies than AD may be contributing to, or entirely responsible for, the cognitive symptoms.

It is expected that individuals who have a worse clinical than biological AD stage would show signs of other brain pathologies, such as Lewy body pathology, cerebral small vessel disease, or limbic-predominant age-related TAR DNA-binding protein 43 (TDP-43) encephalopathy disease, whereas those who perform clinically better than expected based on their biomarkers might have greater resilience to AD pathology.^4,5^ However, it has not yet been tested in the context of the new staging criteria, which was the goal of the current study. We applied the revised clinical and biological (operationalized based on tau-PET) staging systems to participants in the large and deeply phenotyped BioFINDER-2 and Alzheimer Disease Neuroimaging Initiative (ADNI) cohorts, and examined the congruence or discrepancy between their clinical and biological stages.

## Methods

### Participants

#### BioFINDER-2

Participants included individuals from the ongoing prospective Swedish BioFINDER-2 cohort (NCT03174938) that spanned the full spectrum of the AD continuum, ranging from adults with intact cognition or subjective cognitive decline (SCD), mild cognitive impairment (MCI), to dementia.^6^ All participants were recruited in the south of Sweden, were at least 40 years old, and fluent in Swedish. For this particular study, we only included Aβ-positive participants, as required by the biological staging scheme of AD (detailed below). Cognitively intact participants needed to have a Mini-Mental State Examination (MMSE) score of at least 27 (if younger than 66 years) or 26 (if 66 years or older) and no signs of cognitive symptoms as assessed by physicians specialized in cognitive disorders. The cohort also comprised participants with SCD, MCI, or dementia who were all referred to a memory clinic due to cognitive symptoms. Individuals with SCD or MCI had an MMSE score between 24 and 30 and did not fulfill criteria for any dementia, according to the *Diagnostic and Statistical Manual of Mental Disorders* (Fifth Edition) (*DSM-5*). Participants were classified as having MCI if they performed at least 1.5 SDs below the normative score on at least 1 cognitive domain from an extensive neuropsychological test battery,^7^ while participants with SCD performed better than 1.5 SDs. Patients with dementia fulfilled the *DSM-5* criteria for dementia and had an MMSE score of 12 or higher. Clinical diagnosis of AD dementia or other neurodegenerative diseases was determined by experienced clinicians at baseline and reassessed throughout follow-up visits. Further details are provided in the eMethods in Supplement 1. At baseline, medical information related to past or current history of hypertension, hypercholesterolemia, stroke or transient ischemic attack, ischemic heart disease, diabetes, or depression were also recorded. The information was retrieved from both the medical records of each participant and questionnaires answered by the participant and/or their informant. Current or past history of such conditions was coded as 1 and no history was coded as 0. The study was approved by the Swedish Ethical Review Authority and all participants gave written informed consent to participate. Data for the current study was acquired between March 2017 and December 2023. This study followed the Strengthening the Reporting of Observational Studies in Epidemiology (STROBE) reporting guidelines.

#### ADNI

We included Aβ-positive ADNI participants who had a tau-PET scan available. Participants ranged from cognitively normal older adults, patients with MCI, and dementia. Cognitively normal participants had a Clinical Dementia Rating score of 0 and participants with MCI and AD were diagnosed according to standard criteria.^8^ Data included were collected between September 2015 and March 2024.

### Clinical Staging

Clinical staging is operationalized by a 1-to-6 scheme representing clinical severity, which is carried forward largely unchanged from the 2018 research framework and is based on clinical assessment alone. Here, cognitively normal participants were considered as stage 1, SCD as stage 2, MCI as stage 3, and patients with dementia as stages 4 to 6, corresponding to mild, moderate, and severe dementia, respectively. Patients with dementia are not subcategorized by stage in BioFINDER-2, and thus, stages 4 to 6 were analyzed together. In ADNI, as the SCD category was not available, we used the 3 diagnoses available (cognitively normal, MCI, AD dementia).

### Biological Staging

**Figure 1.**
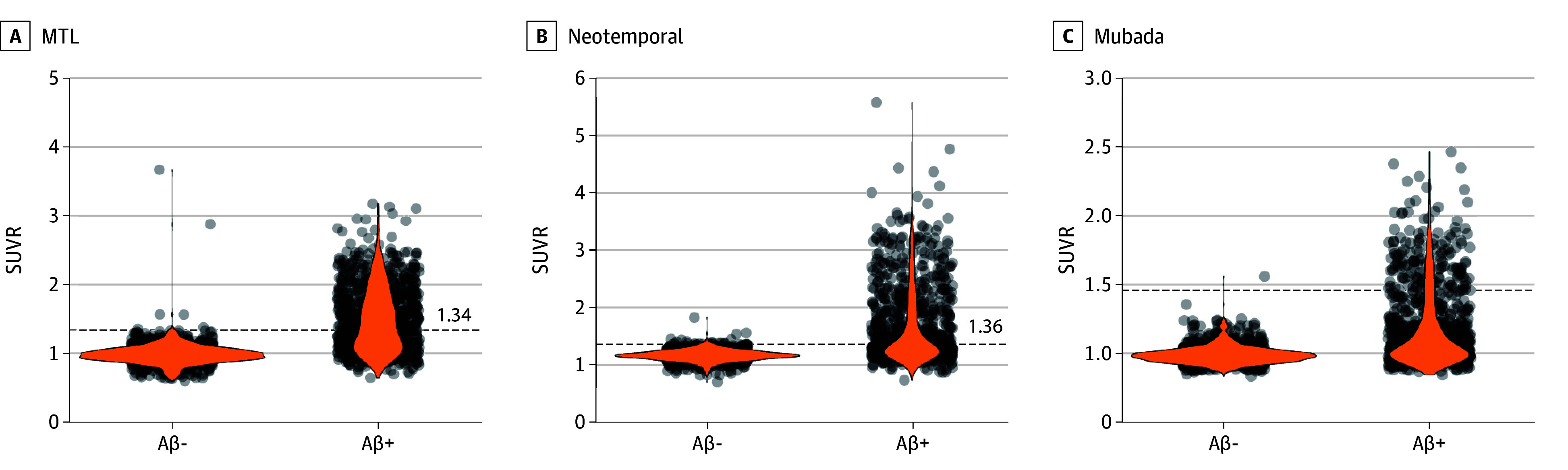
Tau–Positron Emission Tomography Standardized Uptake Value Ratio (SUVR) in the 3 Regions of Interest in BioFINDER-2 The amyloid-β positive group (Aβ+) corresponds to the samples used for the biological stages. The amyloid-β negative group (Aβ−) (n = 1042) is shown to help better understand the cutoffs applied in each cohort. In all panels, the dashed lines represent the cutoff used to determine positivity in each region. MTL indicates medial temporal lobe.

Biological staging was operationalized based on PET, which takes into account the spatial extent of tau-PET (T2 as a core 2 biomarker) and includes 4 stages (A to D). All stages require Aβ positivity (A+). The 4 stages were devised based on recent findings highlighting the importance of the spatial extent of tau accumulation and clinical progression already in cognitively unimpaired older adults:^9,10^ (1) those with elevated tau-PET signal in the medial temporal lobe have a high risk of progressing to MCI over the next few years, unlike those who are only positive on Aβ-PET and (2) those with more advanced tau-PET signal, ie, extending in the neocortex, are at greater risk of progressing to dementia in the same period. Furthermore, in a recent anti-Aβ trial on patients with early symptomatic AD, those with a high tau-PET signal in the neocortex had less clinical benefit of donanemab compared with those with low to intermediate signal.^11^ Given this, the first stage necessitates a negative tau-PET (stage A+T_2_−]); the second is characterized by elevated tau-PET uptake restricted to the medial temporal lobe (MTL) (A+T_2-MTL_+; stage B); the third stage represents a moderate tau-PET uptake in the neocortex, here defined as being tau-positive in the temporal lobe (A+T_2-MOD_+; stage C); and the final stage shows a high neocortical tau-PET signal, here defined as being tau-positive in the temporal lobe and the Mubada region^12^ (A+T_2-HIGH_+; stage D). We included all participants who had Aβ-PET (flutemetamol in BioFINDER-2; florbetaben or florbetapir in ADNI) greater or equal to 20 Centiloids,^13^ or, in the case of patients with dementia in BioFINDER-2, those who were Aβ positive based on CSF Aβ42/40 (patients with dementia do not undergo Aβ-PET in the BioFINDER-2 study). For tau-PET (^18^F-RO948 in BioFINDER-2 and flortaucipir in ADNI), we defined 3 regions to facilitate staging: (1) a medial temporal composite region (T_2-MTL_: average uptake in the entorhinal cortex and amygdala),^9^ (2) a temporal lobe composite region (T_2-MOD_: average uptake in the inferior and middle temporal gyri),^9^ and (3) the Mubada region comprising the neocortex (T_2-HIGH_).^12^ The regions are in line with the supporting evidence on which the biological staging was devised and were previously defined in the literature. Cutoffs were derived in the same way in both cohorts and are described in detail in the eMethods, eTable 1, and eFigure 1 in Supplement 1. Tau-PET standardized uptake value ratio in the 3 regions of interest are shown in Figure 1 (BioFINDER-2) and eFigure 2 (ADNI) in Supplement 1. PET acquisition, processing, and further details on the staging are also explained in Supplement 1.

### Structural Magnetic Resonance Imaging Measures

Different measures related to neurodegeneration were investigated based on T1-weighted, fluid-attenuated inversion recovery, and susceptibility-weighted multigradient echo pulse magnetic resonance imaging (MRI) sequences (Supplement 1 for acquisition parameters). In both cohorts, the T1-weighted images were processed in FreeSurfer version 6.0 and parcellated based on the Desikan-Killiany atlas. We then calculated the average cortical thickness in an AD signature region of interest corresponding to regions of the temporal lobe.^14^ We also calculated a TDP-43–related MRI signature consisting of the ratio between the inferior and middle temporal gyri over the hippocampal volume, where a higher value is indicative of TDP-43 pathology. This temporo-limbic ratio has been proposed as a potential surrogate measure of TDP-43 pathology.^15^ In both cohorts, measures of white matter hyperintensities (WMH) volume, presence of infarcts, and microbleeds (only in BioFINDER-2) were also included. Methodological information for those measures are detailed in the eMethods in Supplement 1.

### Fluid Biomarkers: BioFINDER-2 Only

α-synuclein seed amplification assay in CSF was performed to detect if the sample was positive or negative for α-synuclein aggregates. Assays were performed by the Neuropathology Laboratory at IRCCS-ISNB (Bologna, Italy) with a validated method^16^ and details of the assay have been described previously.^7,17^ Neurofilament light (NfL) and glial fibrillary acidic protein (GFAP) were measured in plasma using the commercially available Simoa kit (Neuro 2-Plex B; Quanterix) at the University of Gothenburg, Sweden. These measurements were done in 2022 and are, therefore, only available for 70% of the population (n = 621).

### Statistical Analyses

The analyses aimed at comparing demographic characteristics (age, sex, education), comorbidities, and markers of neurodegeneration and copathologies between different groups of participants based on their underlying biological and clinical staging. In the main analysis, all participants were categorized into 3 different groups: those with congruent clinical and biological staging (reference group), those whose biological staging was more advanced than their clinical staging (biological > clinical), and those whose biological staging was less advanced than their clinical staging (clinical > biological). In BioFINDER-2, given the large sample size, we conducted supplementary analyses stratified by sex, cognitive status, ie, including cognitively unimpaired (CU) participants (cognitively normal [SCD]) alone, and cognitively impaired (CI) participants (MCI-dementia) alone, as well as when further splitting the sample into 5 groups instead of 3 for comparison, to obtain more granular information. *t* Tests were used for continuous variables and Fisher exact tests for categorical variables. In complementary analyses, markers were also compared between groups when including age and sex as covariates. Post hoc tests were done comparing the different groups to the reference group and we applied false discovery rate (FDR) correction to account for multiple group comparisons for each marker. *P* values with an FDR ≤.05 were considered significant. All analyses and visualizations were done in R version 4.3.2 (R Project).

## Results

### Concordance Between Biological and Clinical Staging in BioFINDER-2

**Figure 2.**
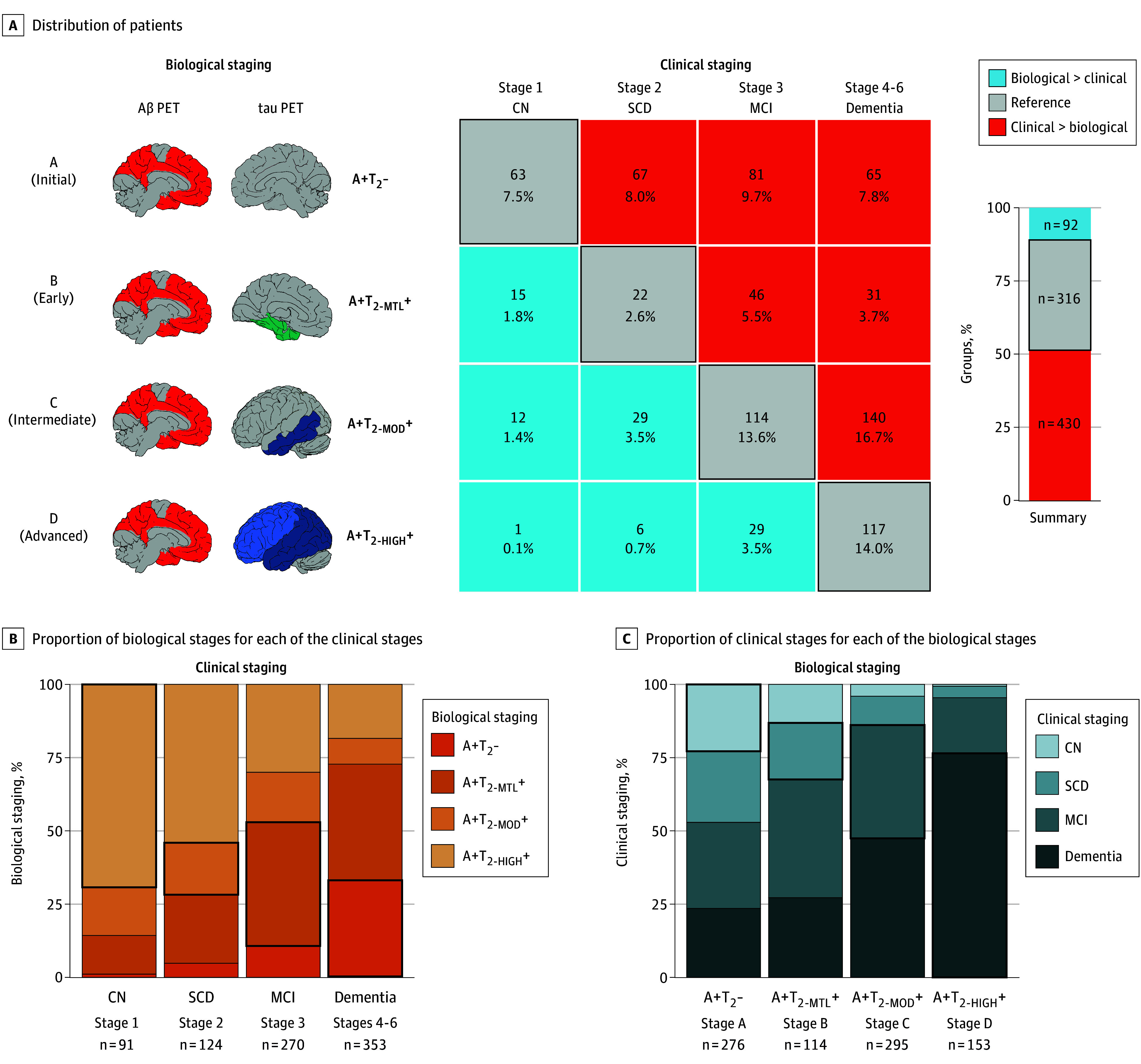
Biological vs Clinical Staging in BioFINDER-2 A, Distribution of participants based on the biological and clinical stages. The numbers and percentages reported are based on the total number of participants. The bar graph shows the overall proportion in the 3 main categories of participants for comparisons. B, Proportion of the 4 biological stages for each of the clinical stages. C, Proportion of the 4 clinical stages for each of the biological stages. In all panels, the black outline represents the cases where the biological and clinical stages are concordant. A+T_2_- indicates amyloid-β positive and tau-positron emission tomography negative; A+T_2-HIGH_+, amyloid-β positive positive and tau-positron emission tomography positive in the neocortex; A+T_2-MOD_+, amyloid-β positive and tau-positron emission tomography positive in the temporal meta region of interest; A+T_2-MTL_+, amyloid-β positive and tau-positron emission tomography positive in the medial temporal lobe; biological > clinical, had more advanced biological impairment compared with their clinical stage; clinical > biological, had more advanced clinical impairment compared with their biological stage; CN, cognitively normal; MCI, mild cognitive impairment; SCD, subjective cognitive impairment.

First, 838 individuals from the BioFINDER-2 cohort were categorized based on clinical and biological staging as operationalized by PET (Figure 2A). All participants were Aβ positive but differed in their tau-PET uptake (T2). There was modest agreement between clinical and biological staging. Specifically, 37.7% of participants exhibited congruency between clinical and biological stages (Figure 2), whereas 51.3% had a clinical stage more advanced than their biological stage (Figure 2). Notably, the largest proportion of participants in this latter group (16.7% of the sample) were patients with dementia and a biological stage of A+T_2-MOD_+ (intermediate biological stage), whereas the concordant biological stage for patients with dementia is A+T_2-HIGH_+ (advanced stage). This large proportion of participants with a more advanced clinical stage than biological stage is in part explained by the presence of Aβ–positive patients with dementia with clinical syndromes other than AD (83 of 353 patients with dementia), which are part of the BioFINDER-2 cohort. This group included 8 participants with frontotemporal dementia, 2 participants with corticobasal syndrome, 5 participants with primary progressive aphasia, 10 participants with vascular dementia, 27 participants with dementia with Lewy bodies, 5 participants with Parkinson disease, 4 participants with progressive supranuclear palsy, and 22 participants with neurodegenerative disorder not otherwise specified. Conversely, participants with a biological stage more advanced than their clinical stage constituted a smaller proportion, 11.0% of the sample (Figure 2). These 3 groups were labeled: reference, biological > clinical, and clinical > biological, and were subsequently characterized based on demographics, comorbidities, and copathologies (Table). The breakdown of participants for each clinical and biological stage is also displayed in Figure 2B and C.

**Table.** Characteristics of the 3 Groups Based on Biological and Clinical Staging in BioFINDER-2

Characteristic	No. (%)					
	Biological > clinical (n = 92)	Reference (n = 316)	Clinical > biological (n = 430)	Group comparisons^a^^,^^b^		
				All^c^	CU^d^	CI^e^
Age, y, mean (SD)	71.8 (8.6)	72.9 (7.9)	75.0 (6.3)	A	A	A,B
Sex						
Female	61 (66.3)	172 (54.4)	198 (46.0)	A	NS	NS
Male	31 (33.7)	144 (45.6)	232 (54)			
Education, y, mean (SD)	13.1 (3.5)	12.9 (4.2)	12.2 (3.8)	A	NS	A
APOE4 carriers	74 (80.4)	227 (71.8)	284 (66.0)	NS	A	NS
α-synuclein positive^f^	9 (10.4)	51 (16.8)	107 (26.8)	A	NS	A
Plasma NfL, pg/ml, mean (SD)^g^	19.2 (7.6)	21.4 (10.8)	25.5 (19.8)	A	NS	A
Plasma GFAP, pg/ml, mean (SD)^g^	210.0 (89.5)	218.8 (113.4)	203.4 (97.5)	NS	NS	NS
Cortical thickness temporal ROI, mm, mean (SD)	2.60 (0.16)	2.51 (0.18)	2.52 (0.20)	B	NS	NS
TDP-43 MRI signature, mean (SD)	5.42 (0.67)	5.34 (0.80)	5.57 (0.79)	A	NS	A
WMH, proportion to ICV, mean (SD)	0.57 (0.45)	0.52 (0.37)	0.63 (0.42)	A	NS	A
Subcortical infarcts	4 (4.4)	15 (4.8)	45 (10.5)	A	NS	A
Cortical and cerebellar infarcts	6 (6.5)	19 (6.0)	42 (9.8)	NS	NS	A
Microbleeds^h^	17 (18.5)	70 (23.1)	91 (22.1)	NS	NS	NS
Hypertension	32 (34.8)	139 (44.0)	181 (42.1)	NS	NS	NS
Hyperlipidemia	24 (26.1)	60 (19.0)	85 (19.8)	NS	NS	NS
Diabetes	12 (13.0)	40 (12.7)	63 (14.7)	NS	NS	NS
Stroke or TIA	6 (6.5)	22 (7.0)	46 (10.7)	NS	NS	NS
Ischemic heart disease	6 (6.5)	25 (7.9)	62 (14.4)	A	NS	A
Depression	12 (13.0)	40 (12.7)	65 (15.1)	NS	NS	NS

Abbreviations: APOE4, apolipoprotein E gene; biological > clinical, had more advanced biological impairment compared with their clinical stage; clinical > biological, had more advanced clinical impairment compared with their biological stage; CI, cognitively impaired; CU, cognitively unimpaired; GFAP, glial fibrillary acidic protein; ICV, intracranial volume; MRI, magnetic resonance imaging; NfL, neurofilament light; NS, no significant differences were found; ROI, region of interest; TDP-43, TAR DNA-binding protein 43; TIA, transient ischemic attack; WMH, white matter hyperintensities.

^a^ All comparisons were done relative to the reference group, with *P* for false discovery rate <.05 considered as significantly different in post hoc tests.

^b^ A indicates the clinical > biological group was significantly different from the reference group and B indicates the biological > clinical group was significantly different from the reference group.

^c^ Refers to the whole sample.

^d^ The CU group includes participants who were cognitively normal or had subjective cognitive impairment.

^e^ The CI group includes patients with mild cognitive impairment and dementia.

^f^ α-synuclein status missing for 49 participants.

^g^ Plasma measures missing for 256 participants.

^h^ Microbleeds assessment missing for 32 participants.

### Comparisons Between the Different Groups in BioFINDER-2

**Figure 3.**
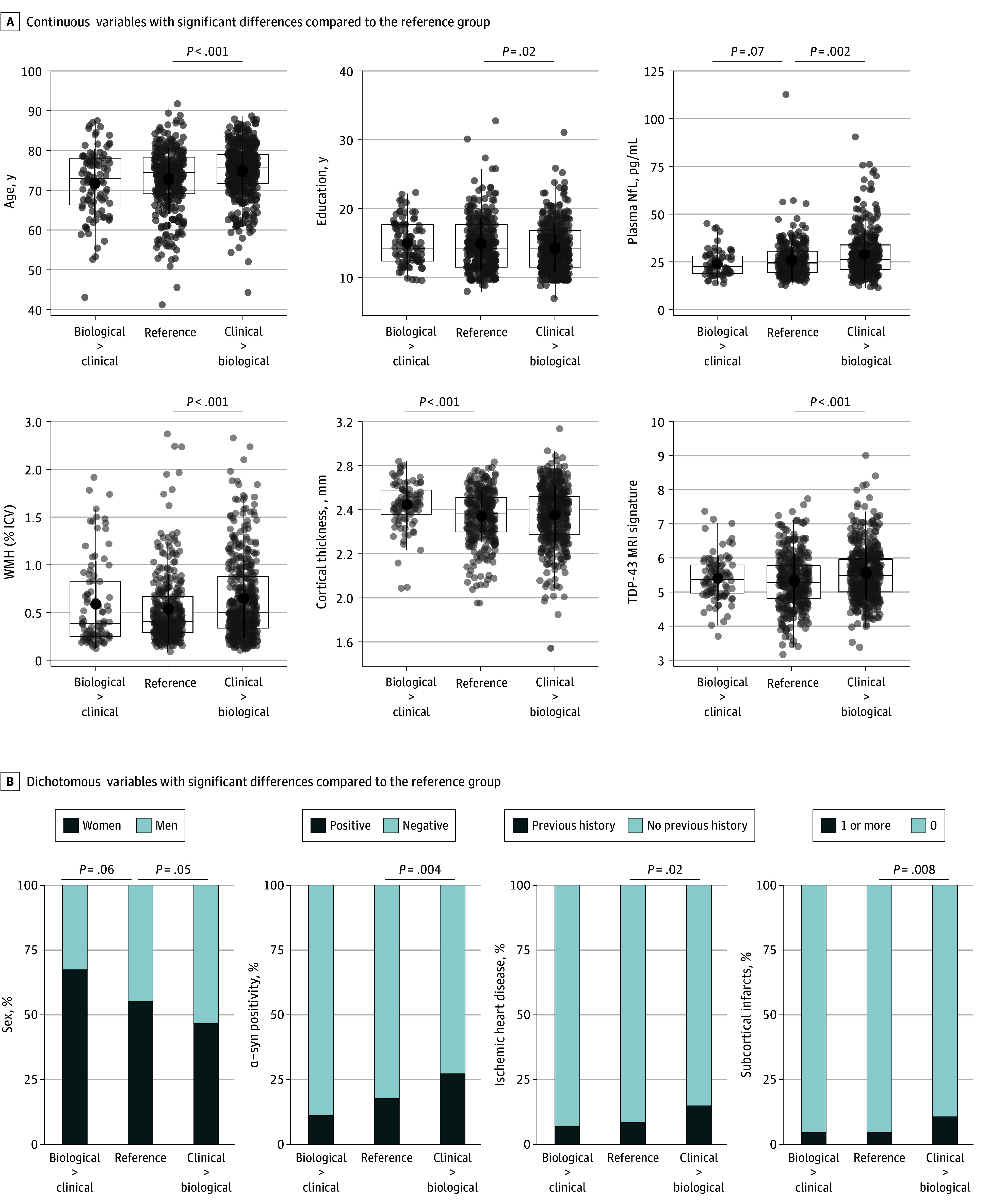
Key Differences Between the 3 Groups, Based on the Biological and Clinical Staging Continuous (A) and dichotomous (B) variables with significant differences compared with the reference group in BioFINDER-2. In panel A, the box limits represent the first and third quartile and the line in the box is the median. All comparisons were done relative with the reference group, with all false discovery rate (FDR) *P* < .05 considered as significantly different in post hoc tests. The all *P* FDR value is reported at the top of each graph, from 2-sided *t* tests for continuous measures and from Fisher tests for binary variables. biological > clinical indicates had more advanced biological impairment compared with their clinical stage; clinical > biological, had more advanced clinical impairment compared with their biological stage; ICV, intracranial volume; Nfl, neurofilament light; WMH, white matter hyperintensities.

Compared with the reference group, the clinical > biological group showed significant differences in several biomarkers and characteristics (Table). Individuals in this group were, on average, 2 years older, included more male individuals (8% more than the reference group), and had on average 1 year less of education than those in the reference group. The most notable differences between these groups were related to neurodegeneration and non–AD pathologies. For example, participants in the clinical > biological group exhibited higher NfL levels, indicating more severe axonal degeneration. They also showed more signs of vascular disease, including more WMH (13% more than the reference group), a higher prevalence of subcortical infarcts, and increased prevalence of ischemic heart disease, with twice as many people with such pathologies than in the reference group. Additionally, this group had a more pronounced MRI signature, suggestive of TDP-43 pathology, and included 10% more participants with α-synuclein pathology than the reference group. Fewer differences were observed between the reference and the biological > clinical group. The only significant distinction was that participants in the latter group showed less neurodegeneration, as indicated by a thicker cortex in the temporal lobe, than the reference group and there was a trend toward having more women (12% more than in the reference group; all FDR *P* = .06). Significant differences between groups are highlighted in Figure 3, along with statistical comparisons.

Accounting for the strong effects of age and sex on several markers of interest, the group comparisons were repeated when including age and sex as covariates (eTable 2 in Supplement 1), as well as stratified by sex (eTable 3 in Supplement 1). Almost all associations remained consistent, with a few differences reduced to trend-level significance when adjusting for age and sex, and certain non–AD markers showing sex-specific associations (all details in the eResults in Supplement 1).

Further analysis was conducted by dividing the sample between CU (eTable 4 in Supplement 1) and CI (eTable 5 in Supplement 1) participants to determine if a similar pattern of differences existed between the 3 groups. Results are summarized in the Table and detailed in the eResults in Supplement 1. Overall, very few differences between groups were observed in the CU group, whereas almost all differences found in the whole sample were also present in the CI group only. Additional analyses were also conducted holding the clinical stage constant in the CI group, specifically comparing extreme groups (initial stage [A+T_2_−] vs the more advanced stages) in MCI and dementia separately (eTable 6 in Supplement 1). These analyses reinforced the findings that copathologies are more present at low levels of AD pathology, as associations were found both in the MCI and dementia groups. It was also found that in the dementia group, participants in the initial biological stage presented more comorbidities (hyperlipidemia, diabetes, stroke) than those in the (concordant) advanced stage.

### Comparisons Between More Granular Groups

**Figure 4.**
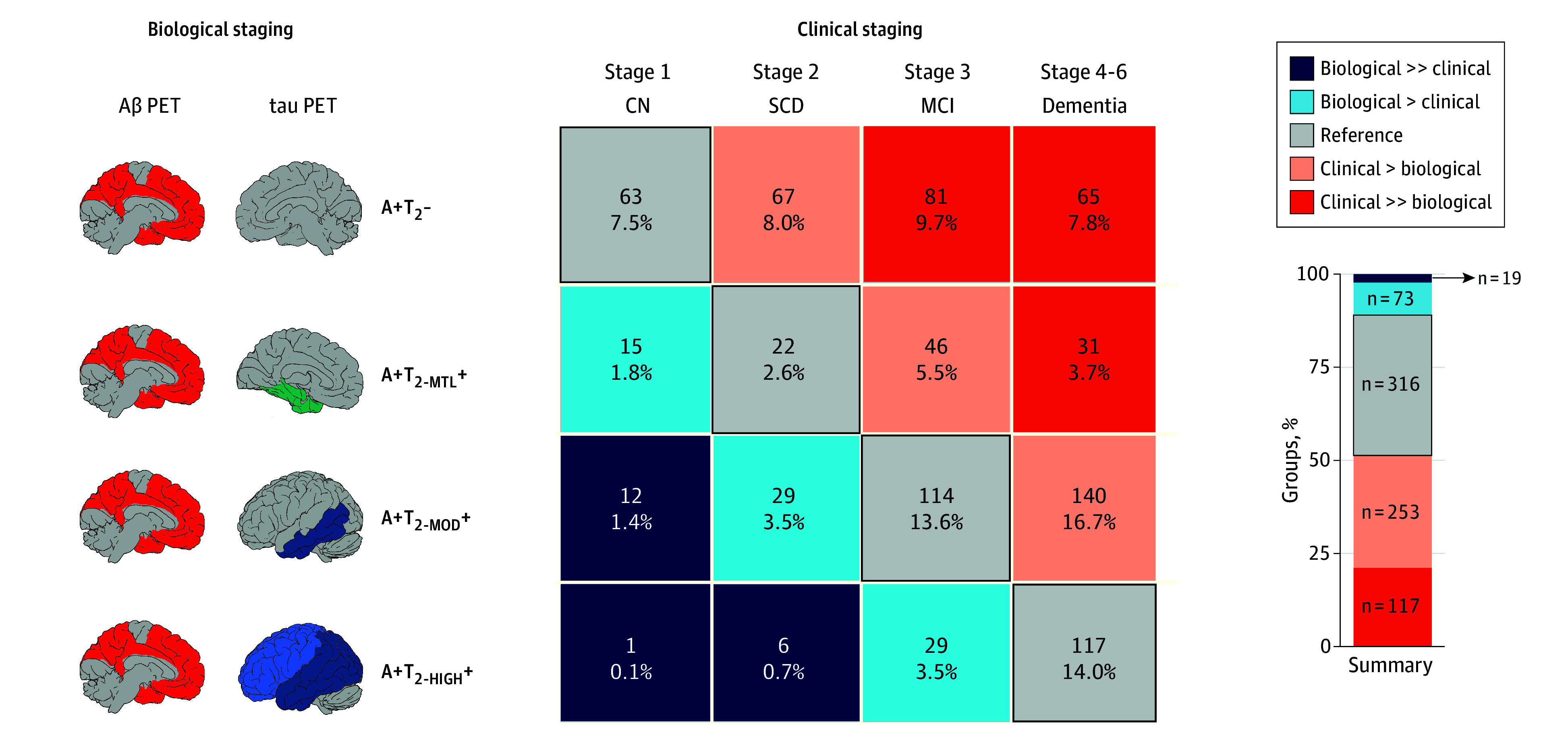
Biological and Clinical Staging Correspondence Split into More Granular Groups The BioFINDER-2 sample was split into 5 groups based on the (dis)congruence of biological and clinical stages, compared with 3 groups, as shown in Figure 1. The bar graph shows the overall proportion in the 5 categories of participants for comparisons. The black outline represents the cases where the biological and clinical stages are concordant. All statistical comparisons and descriptions of the 5 groups are shown in eTable 7 in Supplement 1. A+T_2_- indicates amyloid-β positive and tau-positron emission tomography negative; A+T_2-HIGH_+, amyloid-β positive positive and tau-positron emission tomography positive in the neocortex; A+T_2-MOD_+, amyloid-β positive and tau-positron emission tomography positive in the temporal meta region of interest; A+T_2-MTL_+, amyloid-β positive and tau-positron emission tomography positive in the medial temporal lobe; biological > clinical, had more advanced biological impairment compared with their clinical stage; clinical > biological, had more advanced clinical impairment compared with their biological stage.

The sample was further divided based on the agreement between clinical and biological staging into 5 groups instead of 3 to examine differences between more extreme groups. The reference group remained unchanged, but the other 2 were each split into 2: biological >> clinical, biological > clinical, clinical > biological, and clinical >> biological, as illustrated in Figure 4 (the biological >> clinical and the clinical >> biological groups correspond to those deviating the most from the Reference group). A similar pattern of differences emerged as when the sample was previously split into 3 groups. The 2 groups with more advanced clinical than biological staging showed evidence of higher levels of non–AD brain pathologies (eg, α-synuclein positivity, more advanced TDP-43-like signature, and higher load of WMHs), higher NfL levels, and they were older. All these differences were also observed between the initial 3 groups of the main analyses (Figure 2). At this more granular level, the biggest differences were observed between the biological >> clinical and the reference group, and the demographic differences between groups (sex, education) were attenuated compared with the main analyses. The only different finding compared with the main analyses was that the clinical >> biological group had lower GFAP levels than the reference group (all FDR *P* = .02). The 2 groups with biological stages higher than clinical stages had thicker cortex in the temporal lobe compared with the reference group, indicating signs of resilience to neurodegeneration. No other differences were found in these 2 groups. Detailed characterization of the 5 groups is reported in eTable 7 in Supplement 1.

### Validation in ADNI

In ADNI, 380 individuals were classified based on their biological stage and diagnosis (eFigure 3 in Supplement 1). The agreement between clinical and biological staging was higher than in BioFINDER-2, with 56.1%. There were fewer discordant participants in the clinical > biological group than in BioFINDER-2 with 36.1% and 7.9% of participants were in the biological > clinical group. The groups were very similar if using a slightly less stringent cutoff to define the advanced biological stage (eFigure 4 and eMethods in Supplement 1). Comparing the 3 groups on demographics and several markers also revealed results very consistent with the results in BioFINDER-2 (eTable 8 in Supplement 1). Overall, compared with the reference group, those in the clinical > biological group were, on average, 3.0 years older, included 17% more men, had WMH burden almost twice as high, and an MRI signature more suggestive of TDP-43 pathology (all FDR *P *≤. 01). On the other hand, compared with the reference group, the biological > clinical group had less MRI atrophy, suggestive of TDP-43 pathology (all FDR *P* = .003) and there was a trend toward having more women (all FDR *P* = .08).

## Discussion

In this comprehensive comparison of individuals across the AD continuum stratified according to their biological and clinical stage, we found that those who had a more advanced clinical stage compared with their biological stage exhibited more copathologies and harbored higher levels of neurodegeneration compared with those for whom their clinical and biological stages matched (reference group). Conversely, fewer differences were found in the opposite group, ie, individuals with a biological stage more advanced than their clinical stage, who only showed less cortical atrophy than the reference group. These results highlight that the presence of multiple pathologies may play a predominant role in determining cognitive impairment in people with lower levels of AD pathology than expected compared with their clinical stage.

The revised AD criteria operationalized with PET are not strict regarding which regions should be included in the different tau-PET (T2) stages. Here, we used meta regions previously identified, which yielded a majority of individuals in the reference group (ADNI) or in the clinical > biological group (BioFINDER-2). Given that elevated tau-PET is closely linked to cognitive decline and clinical progression,^18,19^ even in cognitively unimpaired older adults compared with those with only elevated Aβ,^9,10^ it is expected that only a small proportion of the sample (here 11% to 12% in both cohorts) would be resilient to AD pathology (ie, having higher tau-PET levels than expected for their clinical stage). Despite the different compositions of participants in the BioFINDER-2 and ADNI cohorts, results were very consistent, with the clinical > biological groups presenting more non–AD pathologies. ADNI, unlike BioFINDER-2, includes patients with dementia only due to AD and includes less CI participants (59.2% in ADNI vs 74.3% in BioFINDER-2), which together, explains the higher percentage of clinical > biological participants seen in BioFINDER-2. Furthermore, in BioFINDER-2, when splitting the whole sample into 5 groups, we observed that the clinical >> biological group was the one presenting more striking differences compared with the reference group, as can be expected from this more extreme group. While we acknowledge that there are not yet agreed-on criteria to determine the biological stages and different approaches are possible, the current distribution of participants across the different categories with expected proportions across the 4 biological stages in relation to the clinical continuum in 2 cohorts supports the incorporation of the spatial extent of tau-PET into the revised criteria and our current implementation.

We noted that in both cohorts, patients with dementia were largely split between the intermediate biological stage (A+T_2-MOD _+) and the expected advanced stage (A+T_2-HIGH _+). The results suggest that at the more advanced clinical stages, tau-PET levels might be less predictive of the level of cognitive impairment, and we showed that copathologies were more present when the clinical stage exceeded the biological stage. The current in vivo results are in line with the abundant literature based on neuropathology showing that the presence of multiple pathologies is the norm rather than the exception with advanced age and neurodegenerative diseases^20,21^ and recent studies on patients with low tau-PET levels.^22,23^ These results are also important in relation to clinical trials and antiamyloid therapies,^24^ where the presence of copathologies might influence (and lower) the response to drugs targeting AD pathology.

### Strengths and Limitations

The strengths of our study include integrating a large set of markers representing different pathologies and comorbidities in 2 deeply phenotyped cohorts. It represents a first attempt to test the revised criteria in 2 cohorts—a large cohort representative of the general population and memory clinic patients and a volunteer-based cohort focused on AD—and to understand the key factors in the (dis)congruence between clinical and biological staging. A main limitation in both cohorts is that they are not ethnically diverse, which limits the generalizability of the findings. For instance, differences in tau levels, atrophy, and WMH have been reported between different racial and ethnic groups.^25,26^ It will be important to assess the operationalization of the revised criteria in diverse populations and to standardize the operationalization of the stages.^27^ The current work focused on PET, but with ongoing development in T2 blood-based biomarkers, a similar biological staging based on blood biomarkers will be interesting to test and compare in the future.^5^

## Conclusions

We classified 2 large cohorts of participants, covering the AD continuum based on their clinical and biological stages according to the revised AD criteria operationalized by PET. Older adults with more advanced clinical impairment compared with their biological stage (ie, less tau tangle pathology than expected) showed biomarker evidence of more prevalent α-synuclein and TDP-43 copathologies, vascular lesions, and neurodegeneration.

## Notes

Supplement 1. eMethods. eResults. eFigure 1. Correlation and cutoffs between T2 regions eFigure 2. Tau-PET SUVR in the three regions of interest in ADNI eFigure 3. Biological vs Clinical staging in ADNI eFigure 4. Biological and clinical staging with a lower cutoff for the Advanced biological stage in ADNI eTable 1. Tau-PET cutoffs in BioFINDER-2 and ADNI eTable 2. Comparisons of results without covariates and when including age and sex as covariates eTable 3. Main results when stratifying the groups by sex eTable 4. Differences in CU participants only eTable 5. Differences in CI participants only eTable 6. Analyses comparing extreme biological groups stratified by clinical stage (MCI and dementia) eTable 7. Differences in the whole BioFINDER-2 sample split into 5 groups eTable 8. Differences on the three groups based on biological and diagnosis in ADNI

Supplement 2. Nonauthor collaborators

Supplement 3. Data sharing statement
