# Supplementary material for: Evaluation of the Revised Criteria for Biological and Clinical Staging of Alzheimer Disease
Source: JAMA Neurol. 2025 May 19;82(7):666–75. doi: 10.1001/jamaneurol.2025.1100 (PMC12090069; doi:10.1001/jamaneurol.2025.1100)
Supplement: Supplement 1. — eMethods. eResults. eFigure 1. Correlation and cutoffs between T2 regions eFigure 2. Tau-PET SUVR in the three regions of interest in ADNI eFigure 3. Biological vs Clinical staging in ADNI eFigure 4. Biological and clinical staging with a lower cutoff for the Advanced biological stage in ADNI eTable 1. Tau-PET cutoffs in BioFINDER-2 and ADNI eTable 2. Comparisons of results without covariates and when including age and sex as covariates eTable 3. Main results when stratifying the groups by sex eTable 4. Differences in CU participants only eTable 5. Differences in CI participants only eTable 6. Analyses comparing extreme biological groups stratified by clinical stage (MCI and dementia) eTable 7. Differences in the whole BioFINDER-2 sample split into 5 groups eTable 8. Differences on the three groups based on biological and diagnosis in ADNI [file jamaneurol-e251100-s001.pdf]

Supplemental Online Content

Pichet Binette A, Smith R, Salvadó G, et al; for the Alzheimer's Disease Neuroimaging Initiative. Evaluation of the revised criteria for biological and clinical staging of Alzheimer disease. *JAMA Neurol*. Published online May 19, 2025. doi:10.1001/jamaneurol.2025.1100

eMethods.
eResults.
eFigure 1. Correlation and cutoffs between T2 regions
eFigure 2. Tau-PET SUVR in the three regions of interest in ADNI
eFigure 3. Biological vs Clinical staging in ADNI
eFigure 4. Biological and clinical staging with a lower cutoff for the Advanced biological stage in ADNI
eTable 1. Tau-PET cutoffs in BioFINDER-2 and ADNI
eTable 2. Comparisons of results without covariates and when including age and sex as covariates
eTable 3. Main results when stratifying the groups by sex
eTable 4. Differences in CU participants only
eTable 5. Differences in CI participants only
eTable 6. Analyses comparing extreme biological groups stratified by clinical stage (MCI and dementia)
eTable 7. Differences in the whole BioFINDER-2 sample split into 5 groups
eTable 8. Differences on the three groups based on biological and diagnosis in ADNI

This supplemental material has been provided by the authors to give readers additional information about their work.

© 2025 Pichet Binette A et al. *JAMA Neurology*.

eMethods

BioFINDER-2 cohort

The main exclusion criteria included significant unstable systemic illness, neurological or psychiatric illness, alcohol or substance abuse, or refusing lumbar puncture or neuroimaging. Participants were classified as MCI if they performed 1.5 standard deviation below normative scores on at least one cognitive domain from an extensive neuropsychological battery examining verbal, episodic memory, visuospatial, and attention/executive domains. According to the AA criteria[1], all participants with AD dementia needed to be Aβ-positive, assessed by CSF as patients with dementia do not undergo Aβ-PET. Clinical diagnosis of other neurodegenerative diseases was determined according to the main criteria for each disease, as detailed previously[2]. We note that our operationalization of Stage 2 as SCD largely, but not perfectly, overlaps with the original scheme; although cognitive features are the core changes of Stage 2, neurobehavioral changes can also be included in this stage according to the AD criteria, and thus our current Stage 2 definition is more narrow. The revised criteria also include a clinical stage of 0, corresponding to asympotomatic individuals carrying a deterministic gene of AD, and this population is not part of the BioFINDER-2 study. Lastly, plasma NfL and GFAP were analysed instead of CSF as too many participants were missing these measurements in CSF.

ADNI cohort

ADNI is a multi-site study launched in 2003 as a public-private partnership. The primary goal of ADNI has been to test whether serial MRI, PET, other biological markers, and clinical and neuropsychological assessment can be combined to measure the progression of MCI and early AD. For up-to-date information, see www.adni-info.org. We selected all Aβ-positive participants (based on PET) with a flortaucipir tau-PET scan and diagnosis available within 2 years (3 participants had a longer interval but were already Aβ-positive years before the tau-PET scan). We downloaded the tau-PET scans and the closest T1-weighted MRI from the ADNI database, for in-house processing done as in BioFINDER-2. We also downloaded markers related to cerebral small vessel disease similar to BioFINDER-2 from the ADNI database, and kept measures available within maximum two years of tau-PET. Fluid biomarkers were not available, or not available within 2 years of tau-PET for the vast majority of participants in ADNI.

PET acquisition and processing

In BioFINDER-2, Aβ- and tau-PET images were acquired on digital GE Discovery MI scanners. Acquisition for Aβ-PET was done 90-110 min post injection of ~185 MBq [^{18}F]flutemetamol and 70-90 min post injection of ~370 MBq [^{18}F]RO948 for tau-PET. Images were processed as described previously[3]. Briefly, PET images were attenuation corrected, motion corrected, summed and registered to the closest T1-weighted MRI image processed through the longitudinal pipeline of FreeSurfer v6.0. For Aβ-PET, average SUVR was calculated from a global neocortical region of interest including prefrontal, lateral temporal, parietal, anterior cingulate, and posterior cingulate/precuneus.
In ADNI, Centiloids values were used as a measure of Aβ-PET load and were downloaded from the ADNI database. We downloaded the tau-PET scans with realigned and averaged frames and the closest T1-weighted MRI from the ADNI database for further processing (all images available as of November 30, 2024). Flortaucipir tau-PET and T1 images were processed in-house using the same pipeline as in BioFINDER-2.

Tau-PET cutoffs and biological staging

The tau-PET regions of interest (ROI) for the medial temporal lobe (MTL) (entorhinal and amygdala) and the neocortical temporal lobe (inferior and middle temporal gyri) were all described previously and shown to be associated with clinical progression[4]. The region for high tau corresponded to the Mubada region, encompassing larger parts of the cortex, with higher weights assigned to voxels in the posterior and lateral temporal lobe, parietal lobe, association regions of the occipital cortex, and the left hemisphere[5,6]. In both cohorts, for the medial temporal and neocortial temporal ROIs, cutoffs were derived as two standard deviations from the mean of Aβ-negative cognitively unimpaired participants. In BioFINDER-2, such thresholds had been previously defined[4]. For the Mubada region, we used an approach similar to the original study where cutoffs representing low, intermediate and high tau had been proposed. We calculated tertiles in the distribution of tau-positive participants, and used the cutoff of the highest tertile to represent the Advanced biological stage. This approach resulted in a more conservative threshold compared to the two other regions, as the goals was to capture the highest tau-PET levels. With such a threshold, all participants classified as high tau were also positive in the temporal lobe (the region to define the previous biological stage). In BioFINDER-2, the Mubada threshold was 1.47, very close to the threshold proposed by the donanemab trial for high tau of 1.46 SUVR with flortaucipir. We also note that in a head-to-head study that we conducted comparing flortaucipir and RO948[4], values were highly comparable between tracers in the neocortex, with correlations of 0.93-0.96 in the temporal lobe and

larger neocortical regions As such, in line with the literature and as done previously[7], we used 1.46. We also used 1.46 SUVR in ADNI to be consistent with the external cutoff derived in the literature. However, we note that if applying the tertile calculation on the ADNI dataset, the high tau cutoff would be 1.25, as the overall proportion of tau-positive participants is lower than in BioFINDER-2 and that the sample size is smaller. Using the slightly less conservative Mubada threshold of 1.25 in ADNI, we showed that results were unchanged (eFigure 4). The thresholds used in each cohort are reported in eTable 1 and the SUVR distribution in both Aβ-negative and Aβ-positive participants in each cohort are shown in Figure 1 (BioFINDER-2) eFigure 2 (ADNI).

When applying the cutoffs, 5.0% of participants (n=42) in BioFINDER-2 and 3.7% (n=14) in ADNI were tau discordant, such that they were negative in the MTL ROI but positive in the neocortical temporal or Mubada ROI. Most of those cases are very close to the MTL cutoffs and the majority were patients with MCI or dementia (34/42 in BioFINDER-2 and 10/14 in ADNI), who likely have lower MTL SUVR due to atrophy. In the biological staging, those participants were considered Intermediate or Advanced, depending if they were positive only the neocortial temporal ROI or if they were also positive on the Mubada region, respectively.

MRI acquisition (BioFINDER-2)

MRI was performed on a 3 Tesla Siemens MAGNETOM Prisma scanner with a 64-channel head coil (Siemens Healthcare, Erlangen, Germany). Images analyzed in the current study were a T1-weighted anatomical magnetization-prepared rapid gradient echo sequence (MPRAGE; TR = 1900 ms, TE = 2.54 ms, voxel size = 1 × 1 × 1 mm3), a T2-weighted fluid-attenuated inversion recovery sequence (FLAIR; TR = 5000 ms, TE = 393 ms, matching resolution with the T1-weighted image), and a 3D, multi-gradient-echo pulse sequence (TR = 24 ms; TEs = 5.00, 8.80, 12.60, 16.40, and 20.20 ms with monopolar/fly-back readout gradients, flip angle = 15°; voxel size = 0.8 × 0.8 × 1.4 mm3). The 3D multi gradient-echo pulse sequence images with the strongest susceptibility weighting (TE 20.20 ms) were used to assess the number and location of microbleeds in line with established criteria, as used previously[8].

Measures of cerebral small vessel disease

In BioFINDER-2, white matter hyperintensities (WMH) were segmented using the Sequence Adaptive Multimodal SEGmentation (SAMSEG) tool from FreeSurfer v7.1 with the T1 and FLAIR images as input[15]. Infarcts and microbleeds were identified and counted via visual inspection by an experienced neuroradiologist (D.W.), from the FLAIR (infarcts) and the multi gradient-echo pulse sequence (microbleeds) images.
In ADNI, we used WMH volume based on FLAIR and T1 images and infarcts assessment available from the ADNI database.
In both cohorts, for analysis, total WMH volume divided by total intracranial volume was used for analysis. Infarcts were separated into two categories: 1) subcortical infarcts, and 2) cortical and cerebellar infarcts. We used dichotomous variables where for each lesion type, with participants having either no lesion or lesions present.

eResults

Group comparisons adjusting for age and sex or stratified by sex in BioFINDER-2

Most associations remained when adjusting for age and sex (eTable 2); however, the contrasts between the Clinical > Biological group and the Reference group were reduced to trend-level significance for ischemic heart disease (p_{FDR}=0.06), presence of lacunes (p_{FDR}=0.08), lower education (p_{FDR}=0.10), and higher NfL levels (p_{FDR}=0.09). Notably, when including these covariates, the Clinical > Biological group exhibited lower GFAP levels than the Reference group (p_{FDR}=0.05), which was not previously significant (p_{FDR}=0.19). If instead repeating the analyses separately in women and men, results were very similar as in the whole group, with a few associations showing sex-specific effects (eTable 3). The higher proportion participants who had alpha-synuclein pathology, subcortical infarcts in the Clinical > Biological group was only seen in men. The thicker cortex suggestive of resilience to AD pathology in the Biological > Clinical group was only seen in women.

Group comparisons in cognitively unimpaired and impaired participants separately in BioFINDER-2

In CU participants, few differences were observed between the Clinical > Biological group (corresponding to SCD A+T_2- participants) and the Reference group. The former was, on average, 3.5 years younger and had a lower proportion of *APOE4* carriers (all p_{FDR}≤0.03). In the CI group, all but one difference found in the whole sample persisted. The exception was the higher proportion of men in the Clinical > Biological compared to the Reference groups which only reached trend-level significance (p_{FDR}=0.07) in the CI group. Additionally, in CI only, the Clinical > Biological group had a higher proportion of participants with cortical and cerebellar infarcts (p_{FDR}=0.01). Lastly, compared to the Reference group, the Clinical > Biological group was 3.6 years older and the Biological > Clinical was 4.6 years younger (both p_{FDR}<0.001). All characteristics between the three groups in CU and CI are reported in Supplementary Table 2 and 3, respectively.

© 2025 Pichet Binette A et al. *JAMA Neurology*.

eFigure 1. Correlation and cutoffs between T2 regions

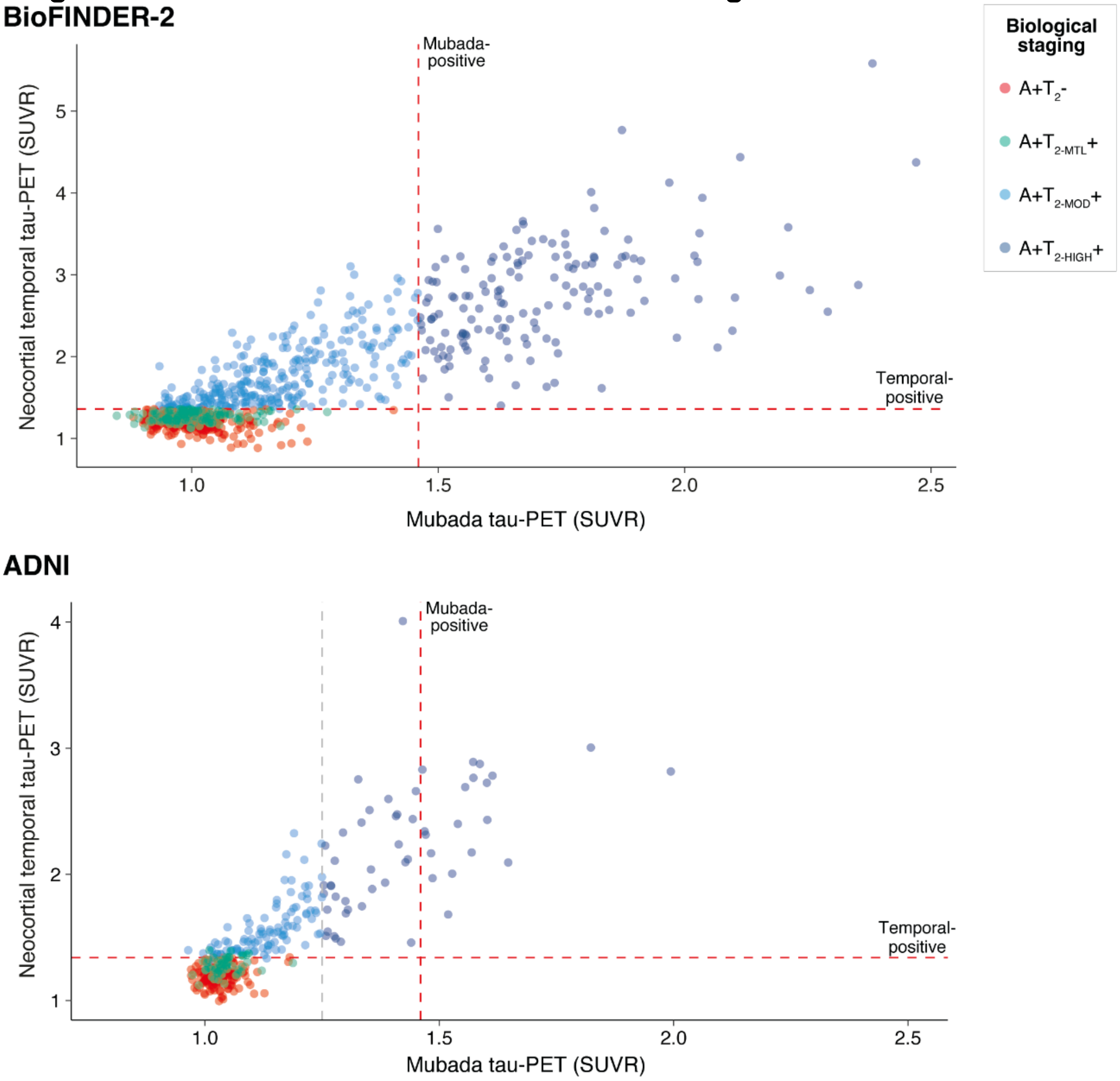

Tau-PET SUVR in the neocortical temporal (y-axis) and Mubada region (x-axis) and the cutoffs used in these two regions shown in red dashed lines in BioFINDER-2 and ADNI. The grey dashed line in ADNI represents the alternative, lower cutoff used in sensitivity analyses.

© 2025 Pichet Binette A et al. *JAMA Neurology*.

eFigure 2. Tau-PET SUVR in the three regions of interest in ADNI

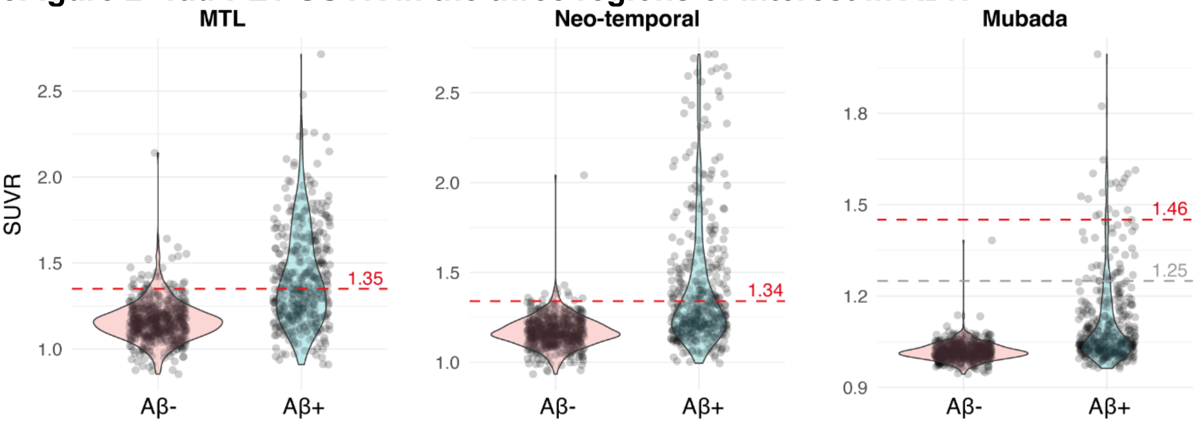

The Aβ-positive group corresponds to the samples used for the biological stages. The Aβ-negative group (n=528) is shown to help better understand the cutoffs applied in each cohort. In all panels, the red dashed lines represent the cutoff used to determine positivity in each region. In the Mubada region, the grey dashed line represents the alternative, lower cutoff, that was used in sensitivity analyses.

© 2025 Pichet Binette A et al. *JAMA Neurology*.

eFigure 3. Biological vs Clinical staging in ADNI

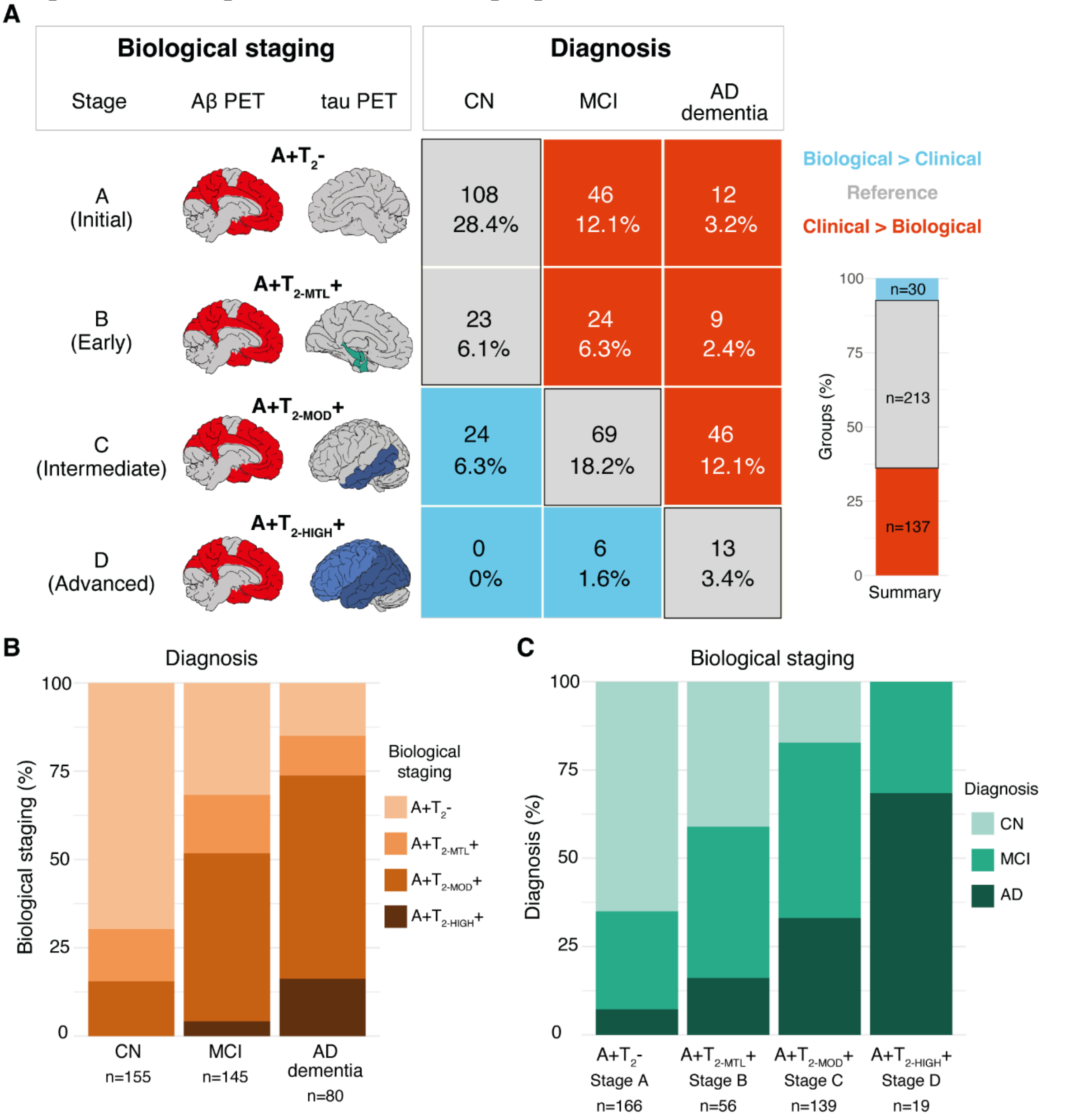

A) Distribution of participants based on the biological stages and diagnosis. The numbers and percentages reported are based on the total number of participants. The bar graph shows the overall proportion in the three main categories of participants for comparisons. The black outline represents the cases where the biological and clinical stages are concordant. B) Proportion of the four biological stages for each of the diagnostic categories. C) Proportion of the three diagnoses in each of the biological stages.

© 2025 Pichet Binette A et al. *JAMA Neurology*.

eFigure 4. Biological and clinical staging with a lower cutoff for the Advanced biological stage in ADNI

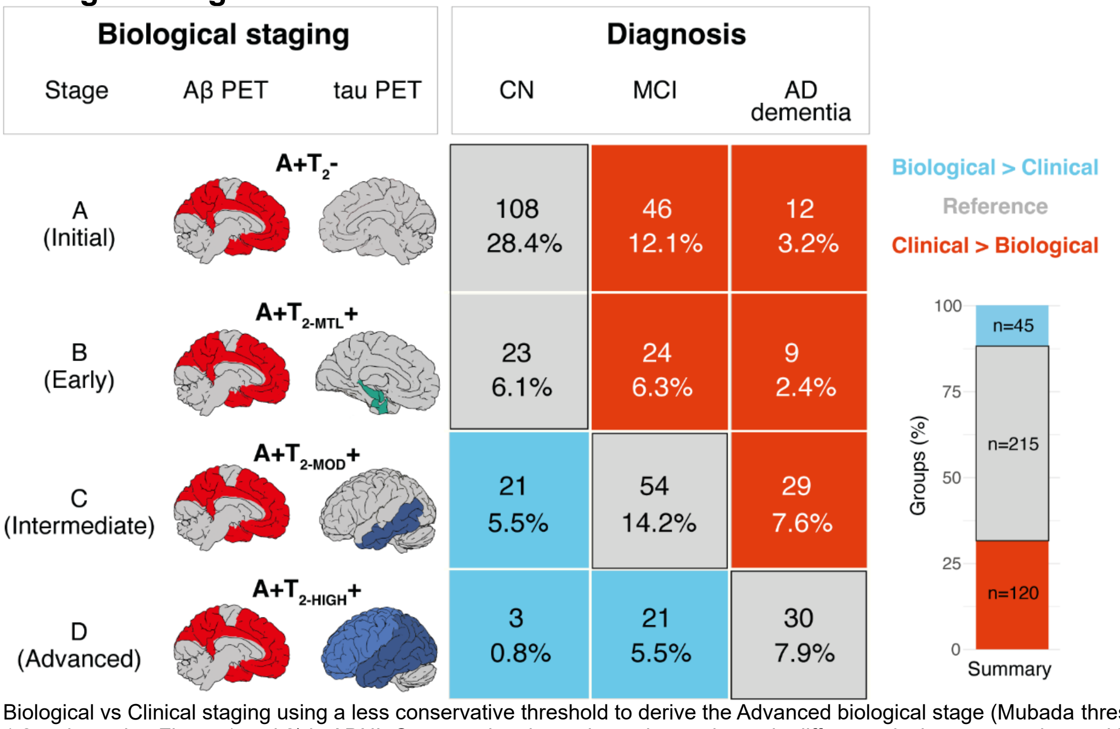

Biological vs Clinical staging using a less conservative threshold to derive the Advanced biological stage (Mubada threshold of 1.25, shown in eFigure 1 and 2) in ADNI. Compared to the main analyses, the main difference is that more patients with Dementia and MCI fell in the Advanced biological stage. Overall, this alternative threshold yielded very similar percentage in the Reference group (56.6%), slightly lower percentage in the Clinical > Biological group (31.6%) and slightly higher percentage in the Biological > Clinical (11.8%). We also note that comparing these three categories yielded consistent results as those shown in eTable 8.

© 2025 Pichet Binette A et al. *JAMA Neurology*.

eTable 1. Tau-PET cutoffs in BioFINDER-2 and ADNI

	**BioFINDER-2 ([^{18}F]RO948)**	**ADNI (Flortaucipir)**	**Approach to derive thresholds**
**Medial temporal lobe**	1.34	1.35	2 standard deviations from the mean of CU Aβ-negative
**Neocortical temporal temporal**	1.36	1.34	
**Mubada**	1.46	1.46	External cutoff derived in the literature with flortaucipir (validated as corresponding to the highest tertile of the tau-positive participants in BioFINDER-2)

All thresholds are SUVR.

© 2025 Pichet Binette A et al. *JAMA Neurology*.

eTable 2. Comparisons of results without covariates and when including age and sex as covariates

	p-value FDR No covariate		p-value FDR Age and sex as covariate	
	Ref vs Biological > Clinical	Ref vs Clinical > Biological	Ref vs Biological > Clinical	Ref vs Clinical > Biological
**Education**	0.71	**0.04**	0.71	0.10
***APOE4* carriers**	0.14	0.14	0.17	0.17
**alpha-syn positive**	0.18	**0.004**	0.20	**0.01**
**Plasma NfL**	0.34	**0.01**	0.50	0.09
**Plasma GFAP**	0.55	0.19	0.44	**0.05**
**Cortical thickness temporal ROI**	**0.001**	0.59	**0.001**	0.11
**TDP-43 MRI signature**	0.36	**<0.001**	0.19	**0.002**
**WMH**	0.31	**0.001**	0.14	**0.02**
**Subcortical infarcts**	1.00	**0.01**	0.92	**0.02**
**Cortical and cerebellar infaracts**	0.81	0.16	0.71	0.26
**Microbleeds,**	0.79	0.79	0.47	0.47
**Hypertension**	0.24	0.65	0.26	0.26
**Hyperlipidemia**	0.29	0.85	0.20	0.96
**Diabetes**	1.00	0.91	0.74	0.74
**Stroke or TIA**	1.00	0.19	1.00	0.37
**Ischemic heart disease**	0.82	**0.02**	0.82	0.06
**Depression**	1.00	0.79	0.81	0.19

Summary p-values (adjusted for FDR) from the main analyses in BioFINDER-2 comparing the Biological > Clinical to the Reference group and the Clinical > Biological to the Reference group in pairwise comparisons (no covariates included in the models) to the same models when including age and sex as covariates. Bolded p-values denote significant result at $p_{FDR} \leq 0.05$.

© 2025 Pichet Binette A et al. *JAMA Neurology*.

eTable 3. Main results when stratifying the groups by sex

	p-value FDR Analyses in women only		p-value FDR Analyses in men only	
	**Ref (n=172) vs Biological > Clinical (n=61)**	**Ref (n=172) vs Clinical > Biological (n=198)**	**Ref (n=144) vs Biological > Clinical (n=31)**	**Ref (n=144) vs Clinical > Biological (n=232)**
**Age**	0.11	**0.05**	0.79	**0.004**
**Education**	0.22	0.21	0.49	0.12
***APOE4* carriers**	1.00	1.0	0.06	**0.04**
**alpha-syn positive**	0.19	0.59	1.00	**<0.001**
**Plasma NfL**	0.37	**0.03**	0.63	0.13
**Plasma GFAP**	0.45	0.33	0.62	0.62
**Cortical thickness temporal ROI**	**<0.001**	0.07	0.36	0.36
**TDP-43 MRI signature**	0.50	**0.006**	0.29	**0.03**
**WMH**	0.92	**0.05**	**0.05**	**0.01**
**Subcortical infarcts**	0.3	0.3	0.15	**0.02**
**Cortical and cerebellar infaracts**	1.00	1.00	0.71	0.23
**Microbleeds**	1.00	1.00	0.50	0.50
**Hypertension**	0.26	0.92	0.84	0.84
**Hyperlipidemia**	0.42	0.42	0.50	0.60
**Diabetes**	0.59	0.44	1.00	1.00
**Stroke or TIA**	0.70	**0.004**	1.00	1.00
**Ischemic heart disease**	0.74	0.09	0.75	0.26
**Depression**	0.83	0.32	1.00	1.00

Summary p-values (adjusted for FDR) from the main analyses in BioFINDER-2 comparing the Biological > Clinical to the Reference group and the Clinical > Biological to the Reference group in pairwise comparisons in women only or men only. Bolded p-values denote significant result at $p_{FDR} \leq 0.05$.

© 2025 Pichet Binette A et al. *JAMA Neurology*.

eTable 4. Differences in CU participants only

	Biological > Clinical (n=63)	Reference (n=85)	Clinical > Biological (n=67)	Group differences (p_{FDR})
**Age, years**	73.9 (7.9)	75.7 (8.4)	72.2 (7.1)	a (0.01)
**Sex, F n (%)**	42 (66.6%)	45 (52.9%)	31 (46.3%)	
**Education, years**	12.7 (3.6)	12.8 (4.2)	12.7 (4.0)	
***APOE4* carriers, n (%)**	51 (81.0%)	64 (75.29%)	38 (56.7%)	a (0.03)
**alpha-syn positive, n (%)**	5 (8.5%)	10 (12.7%)	8 (13.6%)	
**Plasma NfL (pg/mL)[1]**	19.85 (8.05)	19.5 (15.5)	19.5 (9.1)	
**Plasma GFAP (pg/mL) [1]**	211.8 (91.8)	194.8 (116.1)	161.4 (79.5)	
**Cortical thickness temporal ROI (mm)**	2.62 (0.16)	2.65 (0.12)	2.69 (0.14)	
**TDP-43 MRI temporal ratio**	5.44 (0.65)	5.28 (0.62)	5.31 (0.57)	
**WMH (% to ICV)**	0.54 (0.40)	0.44 (0.30)	0.46 (0.35)	
**Subcortical infarcts, n (%)**	3 (4.8%)	3 (3.5%)	6 (9.0%)	
**Cortical and cerebellar infaracts, n (%)**	4 (6.4%)	9 (10.6%)	3 (4.5%)	
**Microbleeds, n (%)[2]**	13 (20.6%)	8 (10.0%)	14 (21.5%)	
**Hypertension, n (%)**	22 (34.9%)	46 (54.1%)	26 (38.8%)	*a (0.07), b (0.06)*
**Hyperlipidemia, n (%)**	14 (22.2%)	22 (25.9%)	13 (19.4%)	
**Diabetes, n (%)**	11 (17.5%)	11 (12.9%)	5 (7.5%)	
**Stroke or TIA, n (%)**	2 (3.2%)	7 (8.2%)	6 (9.0%)	
**Ischemic heart disease, n (%)**	6 (9.5%)	6 (7.1%)	9 (13.4%)	
**Depression, n (%)**	8 (12.7%)	8 (9.4%)	10 (14.9%)	

Data are presented as mean (standard deviation) unless otherwise specicied. All comparisons were done relative to the Reference group, with $p_{FDR} < 0.05$ considered as significantly different in post-hoc tests. Differences are reported in the last column, with the p_{FDR} value in parenthesis. Differences at trend level ($p_{FDR} < 0.1$) are reported in italics. CU participants included correspond to Stage 1 (cognitively normal) and 2 (subjective cognitive decline).
a: Clinical > Biological was significantly different than the Reference group; b: Biological > Clinical was significantly different than the Reference group.
[1] Missing for 82 participants; [2] Missing for 7 participants

© 2025 Pichet Binette A et al. *JAMA Neurology*.

eTable 5. Differences in CI participants only

	Biological > Clinical (n=29)	Reference (n=231)	Clinical > Biological (n=363)	Group differences (p_{FDR})
**Age, years**	67.3 (8.4)	71.9 (7.5)	75.5 (6.1)	a, b (both <0.001)
**Sex, F n (%)**	19 (65.5%)	127 (55.0%)	167 (46.0%)	*a (0.07)*
**Education, years**	13.76 (3.4)	12.9 (4.3)	12.1 (3.8)	a (0.03)
***APOE4* carriers, n (%)**	23 (79.3%)	163 (70.6%)	246 (67.8%)	
**alpha-syn positive, n (%)**	4 (14.8%)	41 (18.3%)	99 (29.0%)	a (0.008)
**Plasma NfL (pg/mL)[1]**	18.1 (6.8)	22.0 (8.9)	26.5 (20.9)	a (0.01)
**Plasma GFAP (pg/mL)[1]**	206.9 (87.2)	226.0 (112.0)	210.5 (98.6)	
**Cortical thickness temporal ROI (mm)**	2.54 (0.16)	2.46 (0.17)	2.49 (0.20)	*a, b (both 0.08)*
**TDP-43 MRI temporal ratio**	5.39 (0.72)	5.36 (0.85)	5.62 (0.82)	a (<0.001)
**WMH (% to ICV)**	0.64 (0.56)	0.55 (0.39)	0.66 (0.43)	a (0.003)
**Subcortical infarcts, n (%)**	1 (3.5%)	12 (5.2%)	39 (10.7%)	a (0.05)
**Cortical and cerebellar infaracts, n (%)**	2 (6.9%)	10 (4.3%)	39 (10.7%)	a (0.01)
**Microbleeds, n (%)[2]**	4 (13.8%)	62 (27.8%)	77 (22.2%)	
**Hypertension, n (%)**	10 (34.5%)	93 (40.3%)	155 (42.7%)	
**Hyperlipidemia, n (%)**	10 (34.5%)	38 (16.5%)	72 (19.8%)	*b (0.08)*
**Diabetes, n (%)**	1 (3.5%)	29 (12.6%)	58 (16.0%)	
**Stroke or TIA, n (%)**	4 (13.8%)	15 (6.5%)	40 (11.0%)	
**Ischemic heart disease, n (%)**	0 (0.0%)	19 (8.2%)	53 (14.6%)	a (0.05)
**Depression, n (%)**	4 (13.8%)	32 (13.9%)	55 (15.2%)	

Data are presented as mean (standard deviation) unless otherwise specicied. All comparisons were done relative to the Reference group, with $p_{FDR} < 0.05$ considered as significantly different in post-hoc tests. Differences are reported in the last column, with the p_{FDR} value in parenthesis. Differences at trend level ($p_{FDR} < 0.1$) are reported in italics. CI participants included correspond to Stage 3 and up (mild cognitive impairment and dementia).
a: Clinical > Biological was significantly different than the Reference group; b: Biological > Clinical was significantly different than the Reference group.
[1] Missing for 174 participants; [2] Missing for 25 participants

© 2025 Pichet Binette A et al. *JAMA Neurology*.

eTable 6. Analyses comparing extreme biological groups stratified by clinical stage (MCI and dementia)

Biological stages	Clinical Stage 3 MCI<br>Initial (n=81) vs Intermediate & Advanced (n=143)	Clinical Stage 4-6 Dementia<br>Initial (n=65) vs Advanced (n=117)	Direction of the results based on the Initial (A+T_2-) group
**Age**	**0.05**	**<0.001**	Older
**Sex**	0.09	**<0.001**	More men
**Education**	0.18	0.32	
***APOE4* carriers**	**0.001**	0.52	Less *APOE4* carriers
**alpha-syn positive**	0.72	**<0.001**	More alpha-syn+
**Plasma NfL**	0.16	**<0.001**	Higher levels
**Plasma GFAP**	**0.04**	**0.03**	Lower levels
**Cortical thickness temporal ROI**	**0.01**	**0.04**	Less atrophy
**TDP-43 MRI signature**	0.07	**0.002**	Signature more suggestive of TDP-43 in dementia
**WMH**	**0.03**	0.06	More WMH
**Subcortical infarcts**	**0.02**	0.12	More infarcts
**Cortical and cerebellar infaracts**	0.23	0.23	
**Microbleeds**	0.52	0.46	
**Hypertension**	0.57	0.64	
**Hyperlipidemia**	0.75	**0.03**	More prevalent
**Diabetes**	0.85	**0.001**	More prevalent
**Stroke or TIA**	0.63	**0.02**	More prevalent
**Ischemic heart disease**	0.07	0.07	
**Depression**	0.83	0.42	

To determine if results in the cognitively impaired participants were due to the severity of clinical stage or can be explained by the levels of AD pathology, we conducted analyses in MCI and dementia groups separately. We compared to most extreme groups, the Initial biological stage to the Advanced stage. Given that in MCI the Advanced biological stage was small (n=29), we combined the Intermediate and Advanced stages. P_{FDR} values are reported in the table, with bolded values being considered significant if $p_{FDR} \leq 0.05$.

© 2025 Pichet Binette A et al. *JAMA Neurology*.

eTable 7. Differences in the whole BioFINDER-2 sample split into 5 groups

	Biological >> Clinical (n=19)	Biological > Clinical (n=73)	Reference (n=316)	Clinical > Biological (n=253)	Clinical >> Biological (n=177)	Group differences (p_{FDR})
**Age, years**	73.7 (8.9)	71.4 (8.5)	72.9 (7.9)	74.5 (6.5)	75.61 (6.0)	a (<0.001), b (0.02)
**Sex, F n (%)**	13 (68.4%)	48 (65.8%)	172 (54.4%)	130 (51.4%)	68 (38.4%)	a (0.003)
**Education, years**	12.0 (3.5)	13.4 (3.5)	12.9 (4.3)	12.1 (3.6)	12.4 (4.1)	*b (0.07)*
***APOE4* carriers, n (%)**	16 (84.2%)	58 (79.5%)	227 (71.8%)	175 (69.2%)	109 (61.6%)	*a (0.06)*
**alpha-syn positive, n (%)**	1 (5.3%)	8 (11.9%)	51 (16.8%)	54 (23.8%)	53 (30.6%)	a (0.003), *b (0.10)*
**Plasma NfL (pg/mL)[1]**	21.9 (8.7)	18.4 (7.1)	21.4 (10.8)	25.0 (22.9)	26.1 (14.4)	a (0.03), b (0.05)
**Plasma GFAP (pg/mL)[1]**	227.8 (73.8)	204.5 (93.7)	218.8 (113.4)	216.2 (102.3)	185.6 (87.7)	a (0.02)
**Cortical thickness temporal ROI (mm)**	2.62 (0.19)	2.59 (0.16)	2.51 (0.18)	2.52 (0.21)	2.52 (0.20)	c (0.009), d (0.03)
**TDP-43 MRI temporal ratio**	5.38 (0.56)	5.43 (0.69)	5.34 (0.80)	5.57 (0.74)	5.57 (0.86)	a (0.002), b (<0.001)
**WMH (% to ICV)**	0.54 (0.39)	0.57 (0.47)	0.52 (0.37)	0.59 (0.40)	0.68 (0.45)	a (<0.001)
**Subcortical infarcts, n (%)**	2 (10.5%)	2 (2.7%)	15 (4.8%)	21 (8.3%)	24 (13.6%)	a (0.003)
**Cortical and cerebellar infaracts, n (%)**	2 (10.5%)	4 (5.5%)	19 (6.0%)	26 (10.3%)	16 (9.0%)	
**Microbleeds, n (%)[2]**	4 (21.1%)	13 (17.8%)	70 (23.1%)	54 (22.1%)	37 (22.0%)	
**Hypertension, n (%)**	5 (26.3%)	27 (37.0%)	139 (44.0%)	106 (41.9%)	75 (42.4%)	
**Hyperlipidemia, n (%)**	4 (21.1%)	20 (27.4%)	60 (19.0%)	48 (19.0%)	37 (20.9%)	
**Diabetes, n (%)**	5 (26.3%)	7 (9.6%)	40 (12.7%)	29 (11.5%)	34 (19.2%)	
**Stroke or TIA, n (%)**	1 (5.3%)	5 (6.9%)	22 (7.0%)	27 (10.7%)	19 (10.7%)	
**Ischemic heart disease, n (%)**	1 (5.3%)	5 (6.9%)	25 (7.9%)	36 (14.2%)	26 (14.7%)	a, b (both 0.04)
**Depression, n (%)**	0 (0.0%)	12 (16.4%)	40 (12.7%)	36 (14.2%)	29 (16.4%)	

Data are presented as mean (standard deviation) unless otherwise specicied. All comparisons were done relative to the Reference group, with $p_{FDR} < 0.05$ considered as significantly different in post-hoc tests. Differences are reported in the last column, with the p_{FDR} value in parenthesis. Differences at trend level ($p_{FDR} \leq 0.1$) are reported in italics. The 5 groups are illustrated in Figure 3.

a: Clinical >> Biological was significantly different than the Reference group; b: Clinical > Biological was significantly different than the Reference group; c: Biological > Clinical was significantly different than the Reference group; d: Biological >> Clinical was significantly different than the Reference group

[1] Missing for 256 participants; [2] Missing for 32 participants

© 2025 Pichet Binette A et al. *JAMA Neurology*.

eTable 8. Differences on the three groups based on biological and diagnosis in ADNI

	Biological > Clinical (n=30)	Reference (n=213)	Clinical > Biological (n=137)	Group differences (p_{FDR})
**Age, years**	73.12 (5.42)	71.70 (6.99)	74.70 (6.97)	a (<0.001)
**Sex, F n (%)**	22 (73.3%)	119 (55.9%)	53 (38.7%)	a (0.004), *b (0.08)*
**Education, years[1]**	16.37 (2.28)	16.26 (2.39)	15.96 (2.59)	
***APOE4* carriers, n (%)[2]**	19 (70.4%)	103 (57.5%)	80 (64.5%)	
**Cortical thickness temporal ROI (mm)**	2.51 (0.14)	2.55 (0.15)	2.51 (0.16)	a (0.05)
**TDP-43 MRI signature**	5.31 (0.48)	5.72 (0.76)	5.99 (0.77)	a (0.003), b (0.005)
**WMH (% to ICV)[3]**	0.69 (0.83)	0.45 (0.65)	0.85 (1.40)	a (0.008)
**Subcortical infarcts, n (%)[4]**	0 (0.0%)	5 (3.1%)	8 (7.8%)	
**Cortical and cerebellar infarcts, n (%)[4]**	3 (13.6%)	19 (11.7%)	11 (10.7%)	

Data are presented as mean (standard deviation) unless otherwise specicied. All comparisons were done relative to the Reference group, with $p_{FDR} \leq 0.05$ considered as significantly different in post-hoc tests. a: the Clinical > Biological group was significantly different from the Reference group; b: the Biological > Clinical group was significantly different from the Reference group. Trend-level associations are reported in italics. The comparisons that were not significant all had $p_{FDR}>0.17$.
[1] Years of education missing for 22 participants
[2] *APOE4* genotype missing for 50 participants
[3] WMH missing for 21 participants
[4] Infarcts count missing for 93 participants

© 2025 Pichet Binette A et al. *JAMA Neurology*.

References

1. Jack CR, Bennett DA, Blennow K, et al. NIA-AA Research Framework: Toward a biological definition of Alzheimer's disease. *Alzheimer's and Dementia*. 2018;14(4):535-562. doi:10.1016/j.jalz.2018.02.018
2. Palmqvist S, Janelidze S, Quiroz YT, et al. Discriminative Accuracy of Plasma Phospho-tau217 for Alzheimer Disease vs Other Neurodegenerative Disorders. *JAMA*. Jul 28 2020;doi:10.1001/jama.2020.12134
3. Leuzy A, Smith R, Ossenkoppele R, et al. Diagnostic Performance of RO948 F 18 Tau Positron Emission Tomography in the Differentiation of Alzheimer Disease From Other Neurodegenerative Disorders. *JAMA Neurol*. May 11 2020;doi:10.1001/jamaneurol.2020.0989
4. Ossenkoppele R, Pichet Binette A, Groot C, et al. Amyloid and tau PET-positive cognitively unimpaired individuals are at high risk for future cognitive decline. *Nat Med*. Nov 2022;28(11):2381-2387. doi:10.1038/s41591-022-02049-x
5. Pontecorvo MJ, Devous MD, Kennedy I, et al. A multicentre longitudinal study of flortaucipir (18F) in normal ageing, mild cognitive impairment and Alzheimer's disease dementia. *Brain*. Jun 1 2019;142(6):1723-1735. doi:10.1093/brain/awz090
6. Devous MD, Sr., Joshi AD, Navitsky M, et al. Test-Retest Reproducibility for the Tau PET Imaging Agent Flortaucipir F 18. *J Nucl Med*. Jun 2018;59(6):937-943. doi:10.2967/jnumed.117.200691
7. Mattsson-Carlgren N, Collij LE, Stomrud E, et al. Plasma Biomarker Strategy for Selecting Patients With Alzheimer Disease for Antiamyloid Immunotherapies. *JAMA Neurol*. Jan 1 2024;81(1):69-78. doi:10.1001/jamaneurol.2023.4596
8. Coomans EM, van Westen D, Binette AP, et al. Interactions between vascular burden and amyloid-beta pathology on trajectories of tau accumulation. *Brain*. Mar 1 2024;147(3):949-960. doi:10.1093/brain/awad317

© 2025 Pichet Binette A et al. *JAMA Neurology*.
