# Supplementary material for: Evaluation of the Revised Criteria for Biological and Clinical Staging of Alzheimer Disease
Source: JAMA Neurol. 2025 May 19;82(7):666–75. doi: 10.1001/jamaneurol.2025.1100 (PMC12090069; doi:10.1001/jamaneurol.2025.1100)
Supplement: Supplement 3. — Data sharing statement [file jamaneurol-e251100-s003.pdf]

# Data Sharing Statement

Pichet Binette. Evaluation of the Revised Criteria for Biological and Clinical Staging of Alzheimer Disease. *JAMA Neurol*. Published May 19, 2025. doi:10.1001/jamaneurol.2025.1100

**Data**
**Data available:** No

**Additional Information**
**Explanation for why data not available:** Pseudononymized data will be shared by request from a qualified academic investigator for the sole purpose of replicating procedures and results presented in the article, and as long as data transfer is in agreement with EU legislation on the general data protection regulation and decisions by the Ethical Review Board of Sweden and Region Skåne, which should be regulated in a material transfer agreement.
